# Author Correction: piRNA processing by a trimeric Schlafen-domain nuclease

**DOI:** 10.1038/s41586-024-07938-4

**Published:** 2024-08-14

**Authors:** Nadezda Podvalnaya, Alfred W. Bronkhorst, Raffael Lichtenberger, Svenja Hellmann, Emily Nischwitz, Torben Falk, Emil Karaulanov, Falk Butter, Sebastian Falk, René F. Ketting

**Affiliations:** 1 Biology of Non-coding RNA group, Institute of Molecular Biology, Mainz, Germany; 2 International PhD Programme on Gene Regulation, Epigenetics & Genome Stability, Mainz, Germany; 3 Max Perutz Labs, Vienna Biocenter Campus (VBC), Vienna, Austria; 4 Department of Structural and Computational Biology, Center for Molecular Biology, University of Vienna, Vienna, Austria; 5 Quantitative Proteomics group, Institute of Molecular Biology, Mainz, Germany; 6 Bioinformatics Core Facility, Institute of Molecular Biology, Mainz, Germany; 7 Institute of Developmental Biology and Neurobiology, Johannes Gutenberg University, Mainz, Germany; 8 Present Address: Institute of Molecular Virology and Cell Biology, Friedrich Loeffler Institute, Greifswald, Germany

**Keywords:** RNAi, RNA, X-ray crystallography, Non-coding RNAs, Germline development

Correction to: *Nature* 10.1038/s41586-023-06588-2 Published online 27 September 2023

After our manuscript was published, we were alerted by an anonymous reader that four elemental ions modeled in the crystal structure of the TOFU-6^eTUDOR/^TOFU-1^pep^ complex are most likely incorrect. Moreover, the density of TOFU-1^pep^ was weak, probably due to low occupancy. To address those issues, we have now determined the structure from a different crystal, diffracting to 2.2 Å, showing clearer density for TOFU-1^pep^. Also, the erroneously modeled elemental ions were corrected. A new version of Supplementary Data has been uploaded containing an updated version of Extended Data Table 1 | Data collection and refinement statistics.

In the main text of the manuscript, we now changed the following sentences:

The original sentence “We determined the crystal structure of the TOFU-6^eTUDOR^–TOFU-1^pep^ complex at a resolution of 1.7 Å (Fig. 5c and Extended Data Table 1)” now reads “We determined the crystal structure of the TOFU-6^eTUDOR^–TOFU-1^pep^ complex at a resolution of 2.2 Å (Fig. 5c and Extended Data Table 1)”.

In the Methods section, the sentence “The refined model has a clashscore of 5.06 and 98.54% of the residues fall into Ramachandran-favoured and 1.46% into Ramachandran-allowed regions” has been updated to “The refined model has a clashscore of 1.17 and 98.56% of the residues fall into Ramachandran-favoured and 1.44% into Ramachandran-allowed regions”.

In the Methods and Data availability sections, the sentence “Coordinates and structure factors of the TOFU-6^eTUDOR^–TOFU-1^pep^ complex structure have been deposited at the PDB (8BY5)” now reads “Coordinates and structure factors of the TOFU-6e^TUDOR^–TOFU-1^pep^ complex structure have been deposited at the PDB (9G6Z)”.

None of the manuscript’s interpretations are affected by this correction. These changes have been made to the HTML and PDF versions of the article.

