# Supplementary material for: piRNA processing by a trimeric Schlafen-domain nuclease
Source: Nature. 2023 Sep 27;622(7982):402–9. doi: 10.1038/s41586-023-06588-2 (PMC10567574; doi:10.1038/s41586-023-06588-2)

nature portfolio
https://doi.org/10.1038/s41586-023-06588-2

Supplementary information

piRNA processing by a trimeric Schlafen-domain nuclease

In the format provided by the authors and unedited

Figure 2A_Raw image files

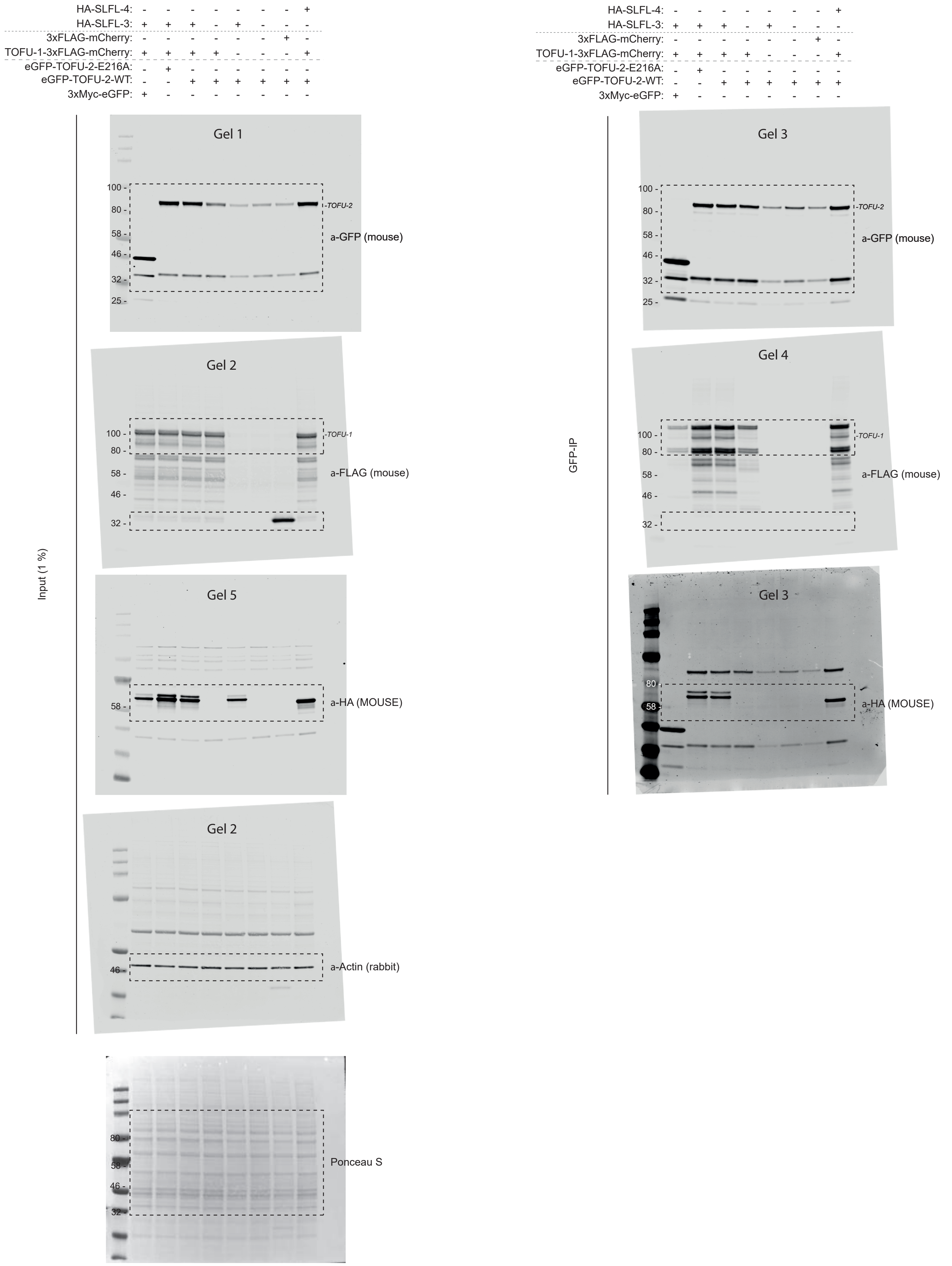

Figure 3b_Raw image files

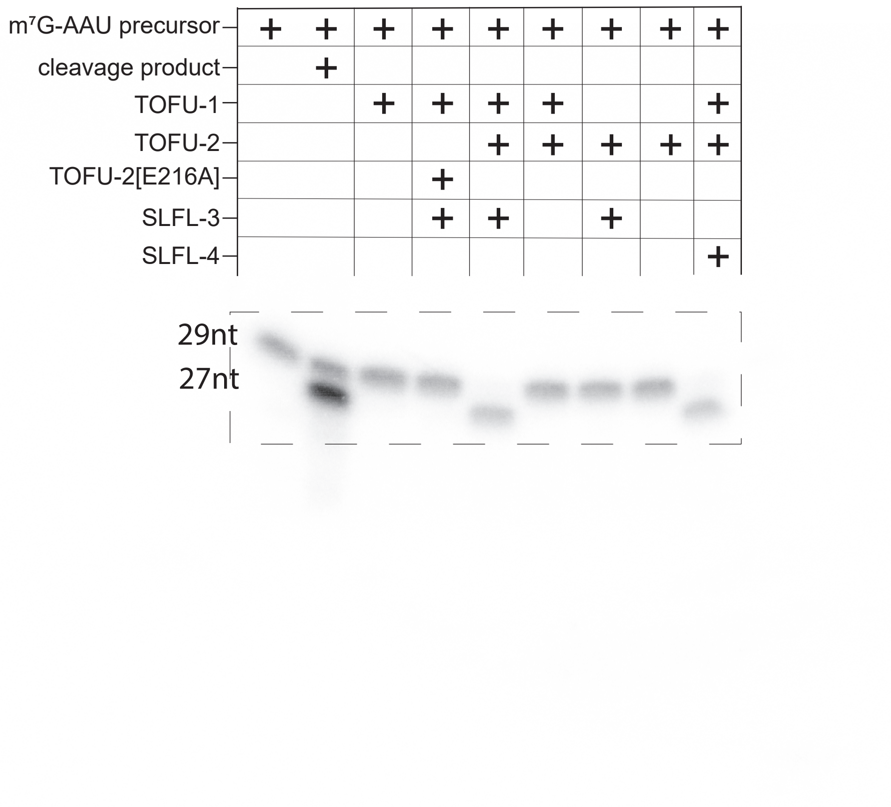

Figure 3c_Raw image files

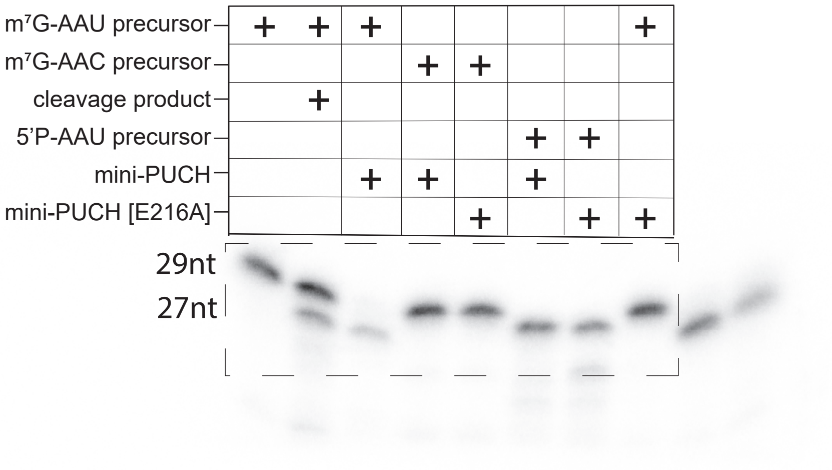

Figure 3d_Raw image files

	1	2	3	4
m⁷G-AAU precursor	+	+	+	+
cleavage product	+			
10-nt RNA oligo			+	+
mini-PUCH			+	
mini-PUCH [E216A]				+

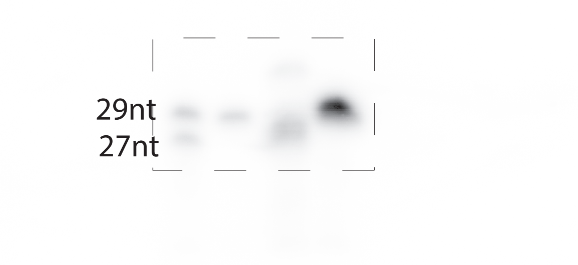

Figure 3e_Raw image files

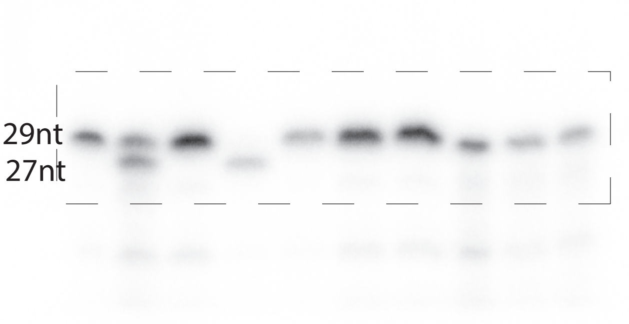

Figure 3f_Raw image files

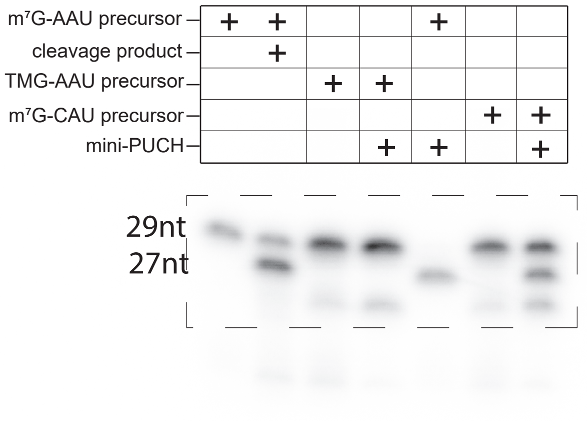

Figure 3g_Raw image files

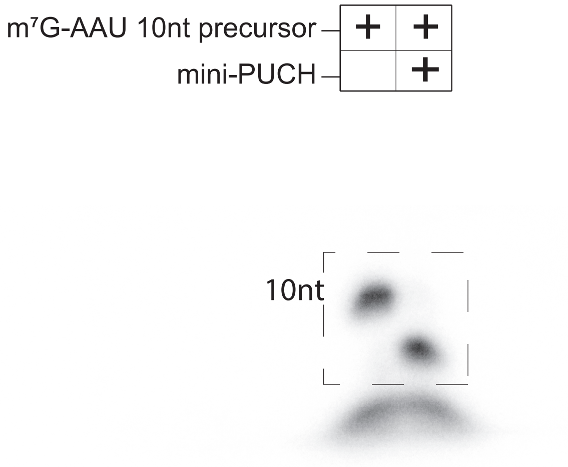

With PETISCO

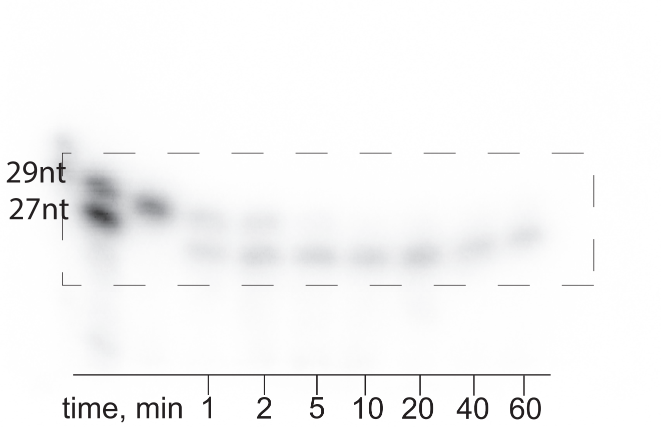

With PETISCO

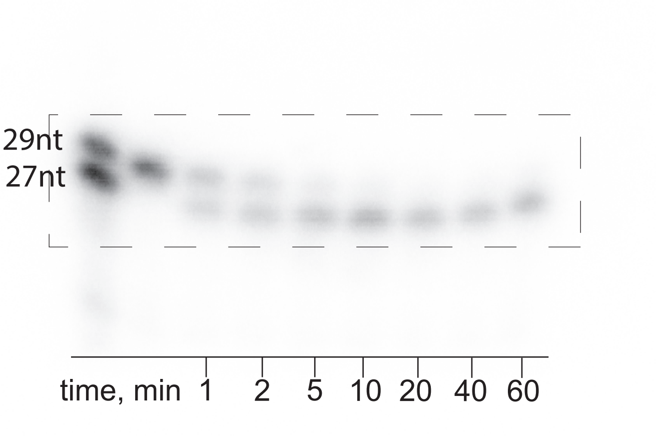

Without PETISCO

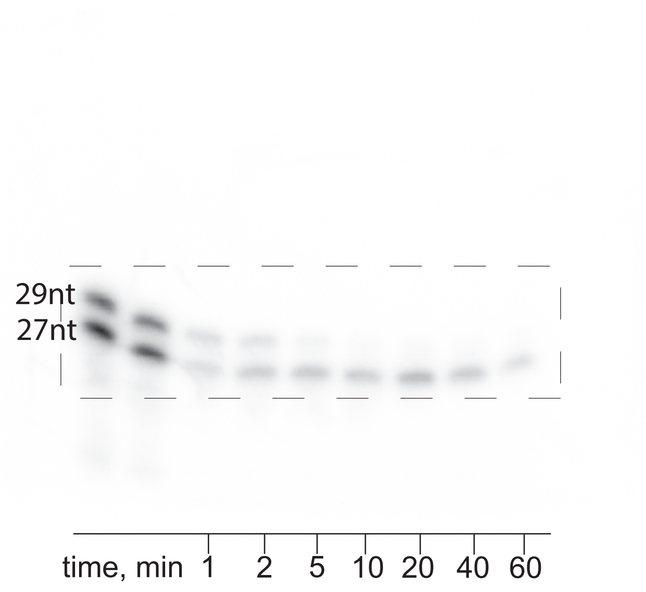

Without PETISCO

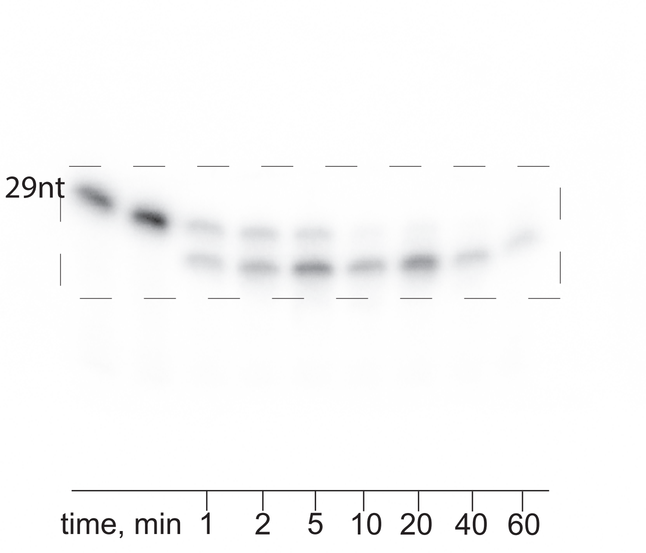

Figure 5a raw data

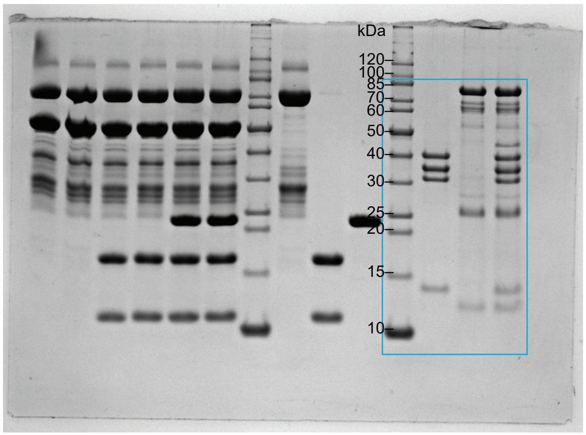

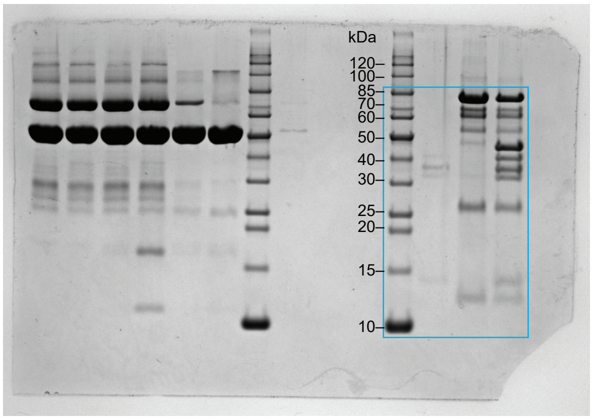

Everything had been run on the same gel, transfered together, membrane was cut to incubate with different primary antibodies, TOFU-6 and TOFU-1 membranes were suparated before imagine

Figure5d_Raw data

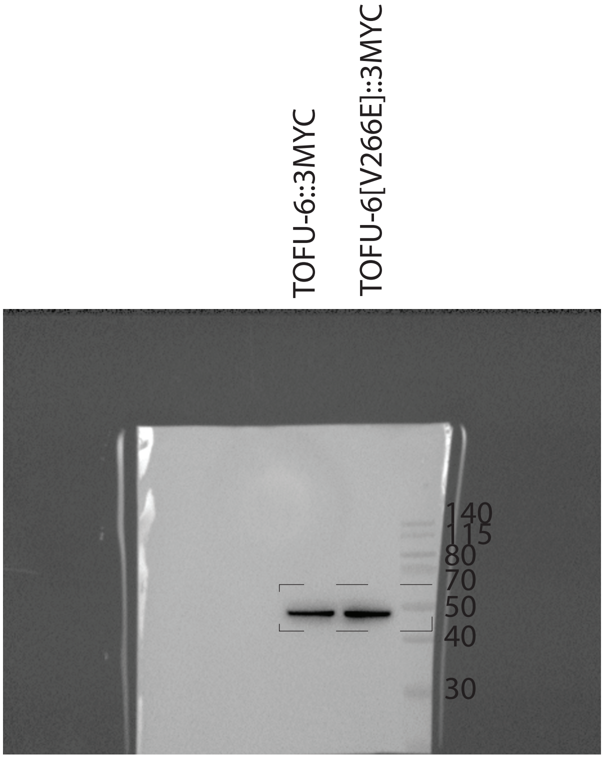

anti-MYC

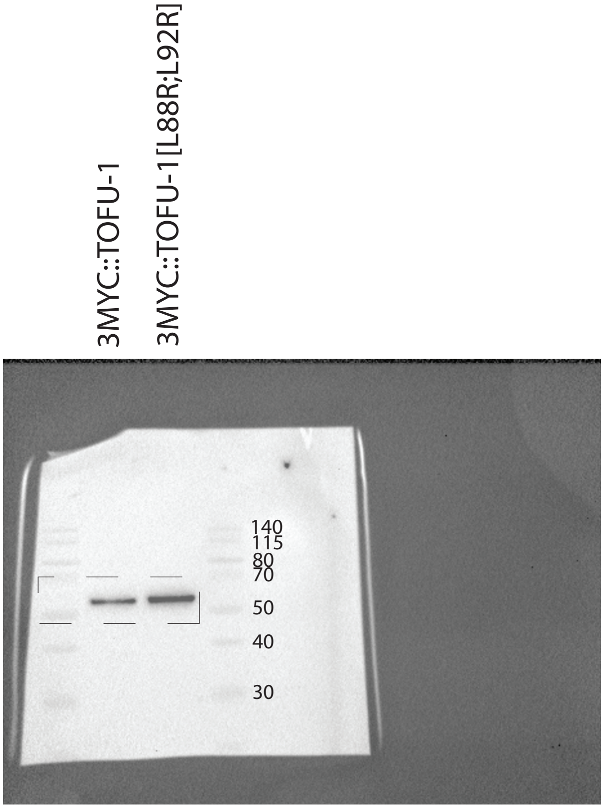

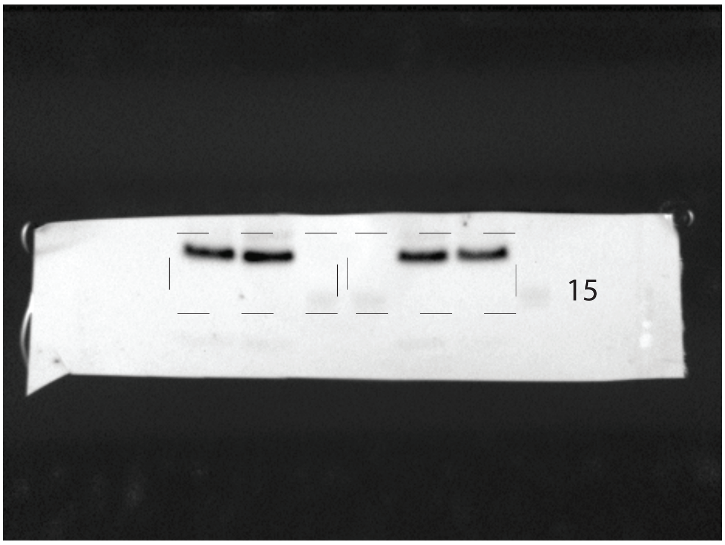

anti-H3

Extended data Figure 1c_Raw data

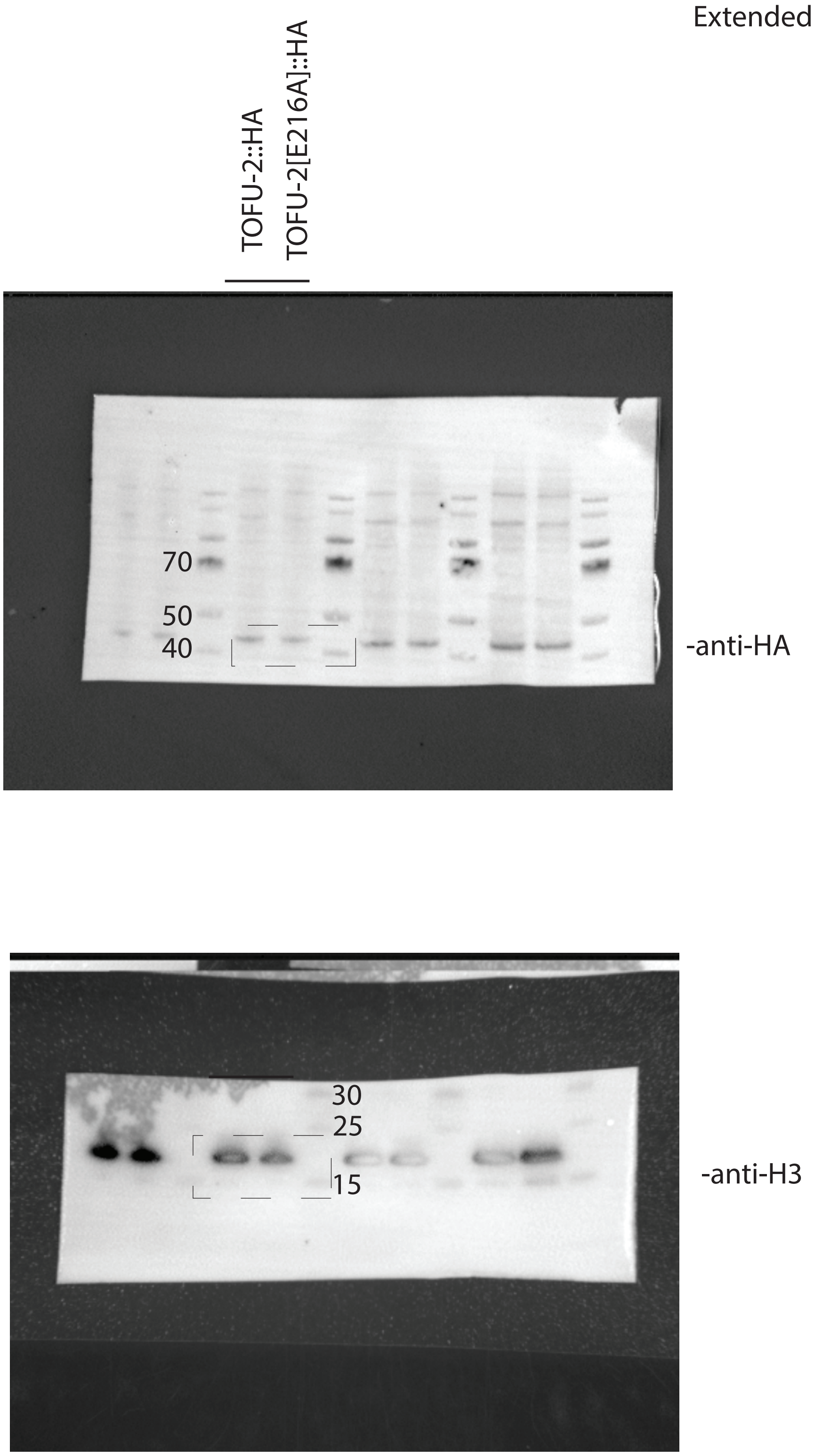

Extended data Figure 5b

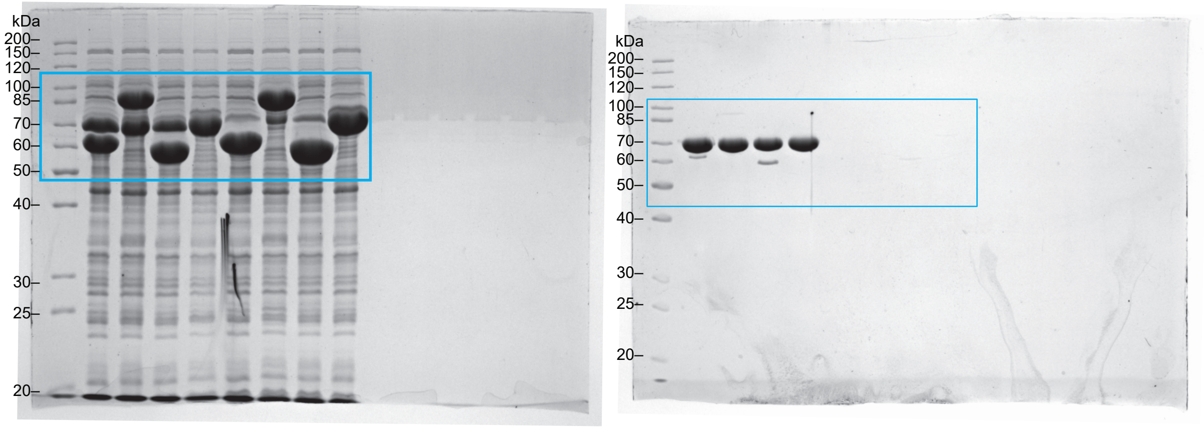

Extended data Figure 5c

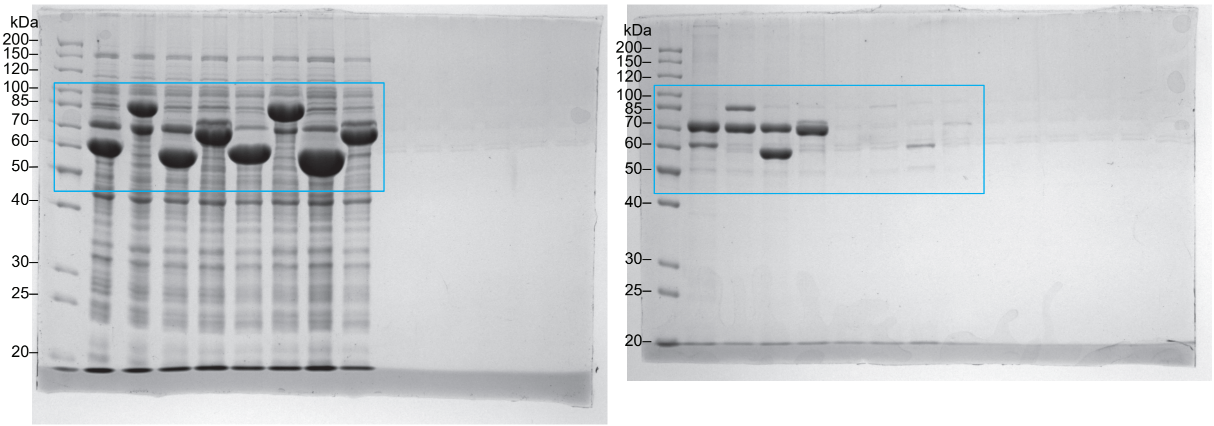

Extended data Figure 5d

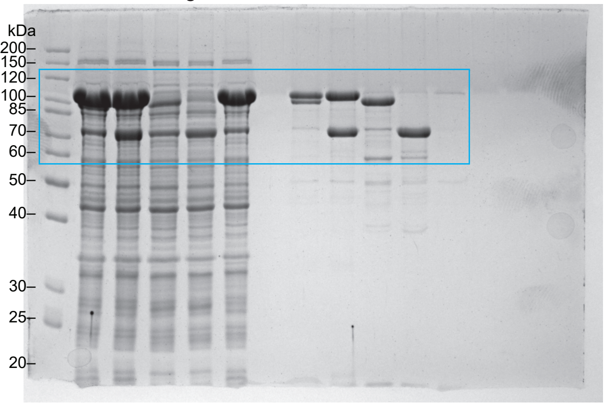

Extended data Figure 5f

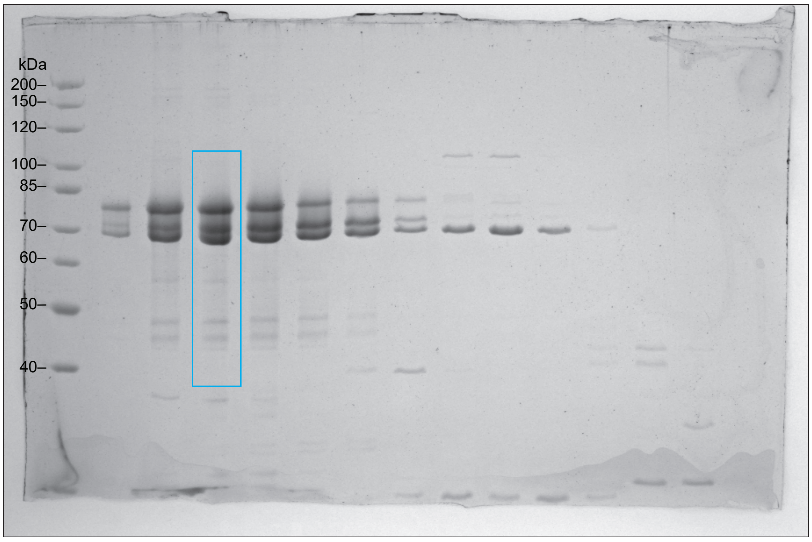

Extended data Figure 5e

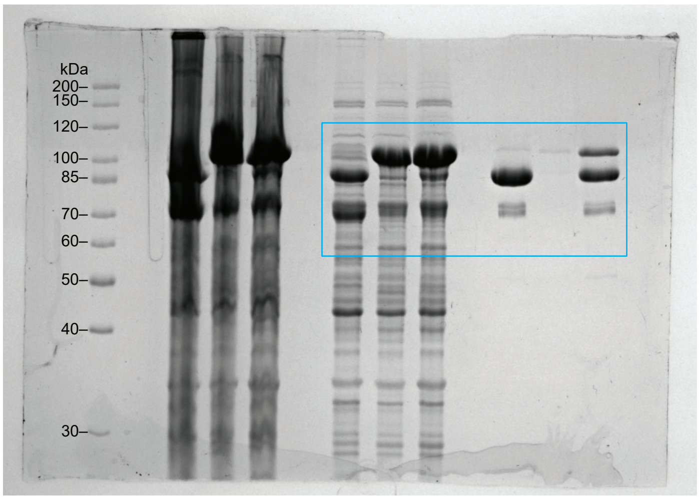

Extended data Figure 5g

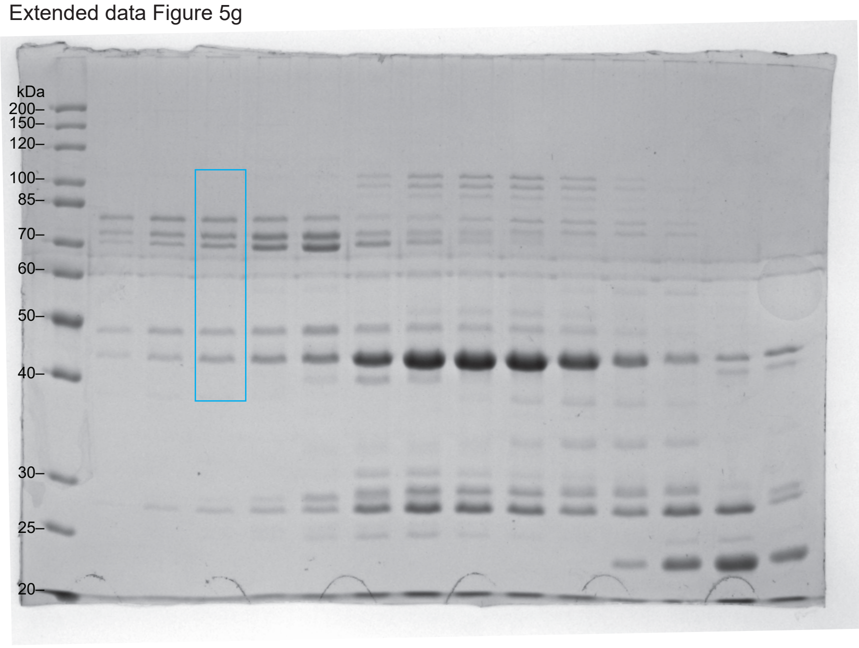

Extended data Figure 6 ab raw data

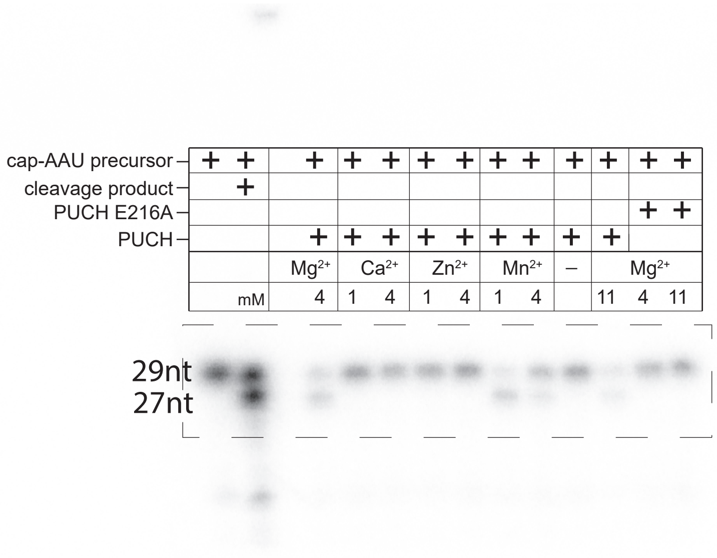

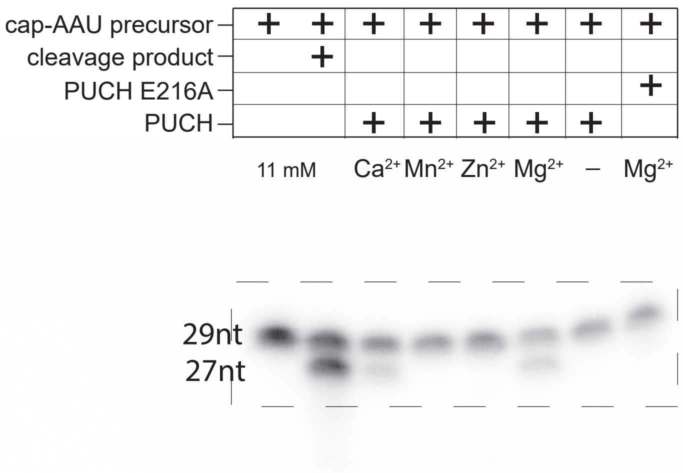

AAU substrate

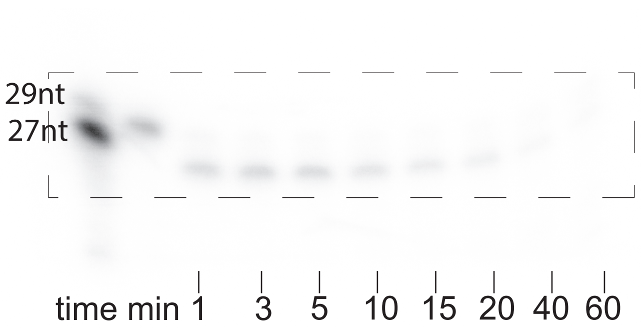

RNA control

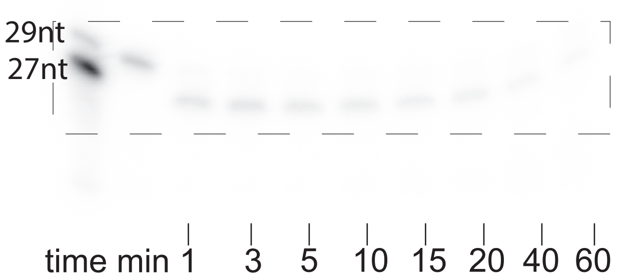

AAU

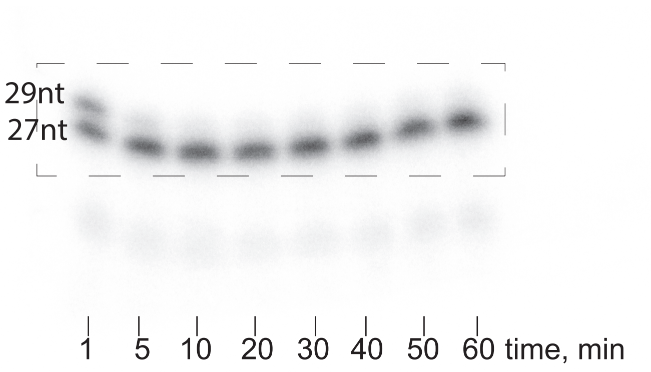
29nt
27nt
1
5
10
20
30
40
50
60
time, min

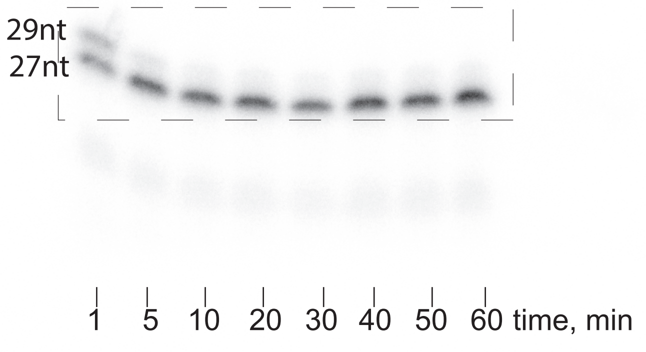
29nt
27nt
1
5
10
20
30
40
50
60
time, min

Extedned data 6d-1 Raw data

CAU substrate

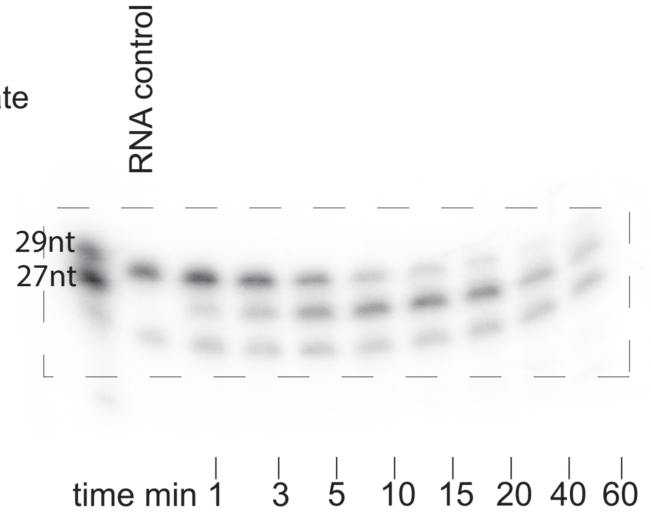

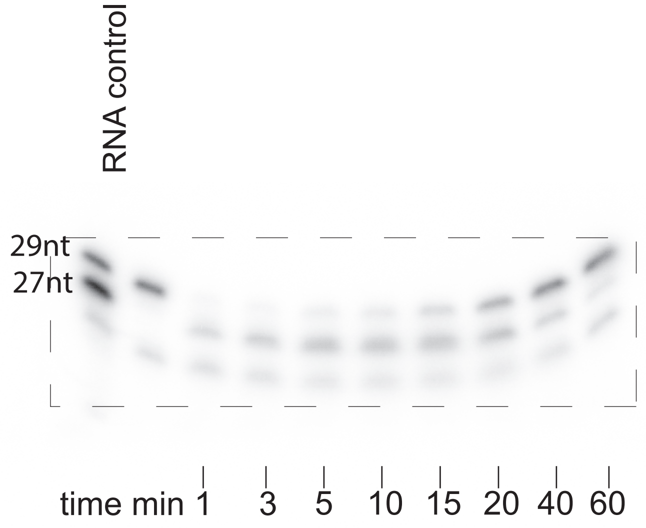

CAU-substrate

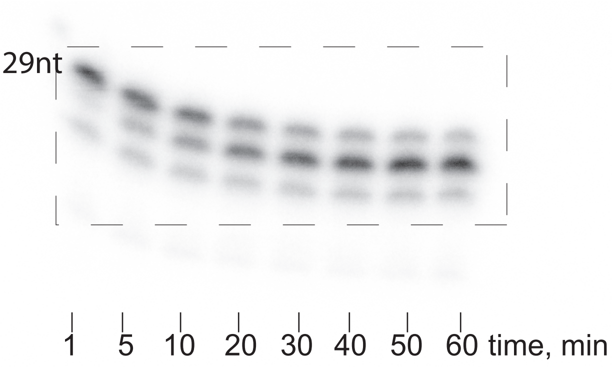

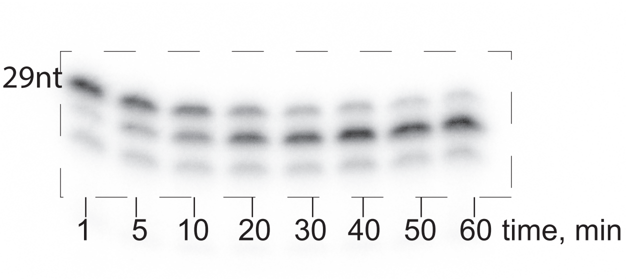

Exteneded data figure 6_f raw data

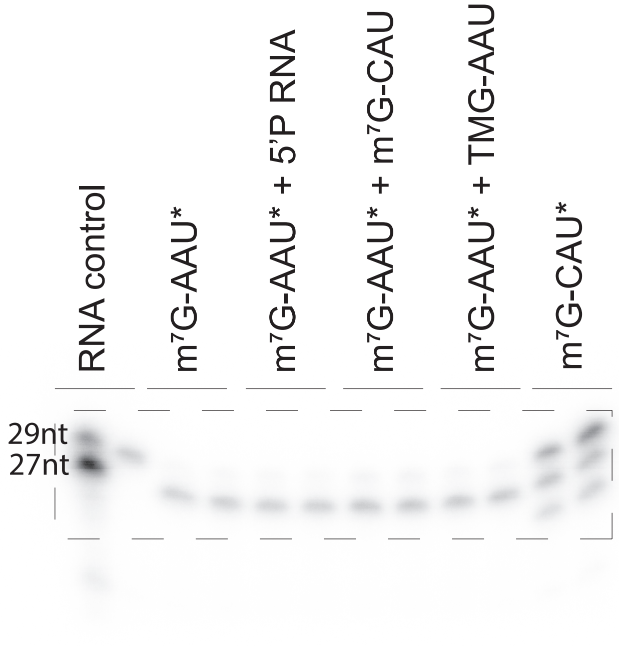

Extened data 6g-1 Raw data

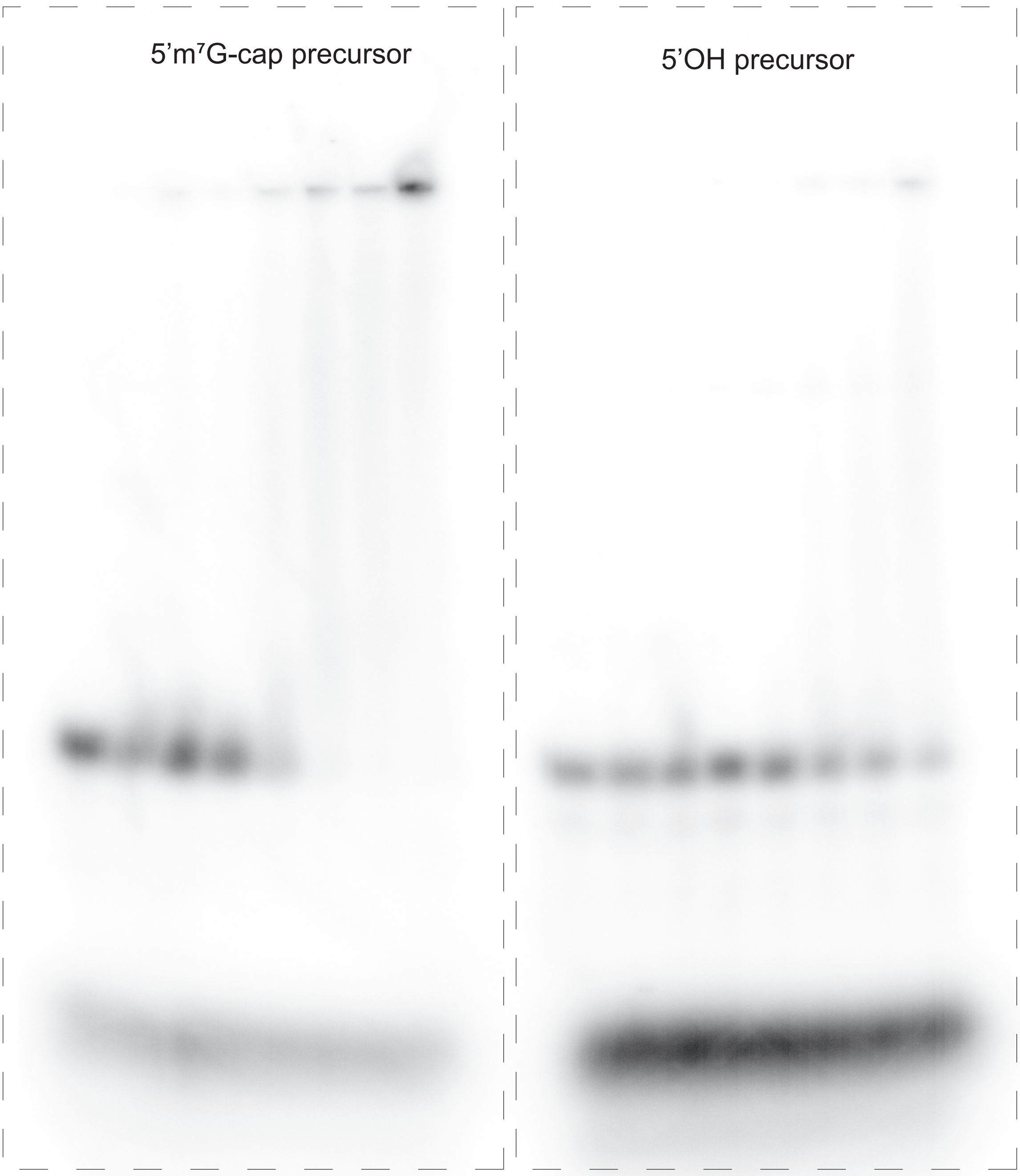

Extended data 6g-2Raw Data

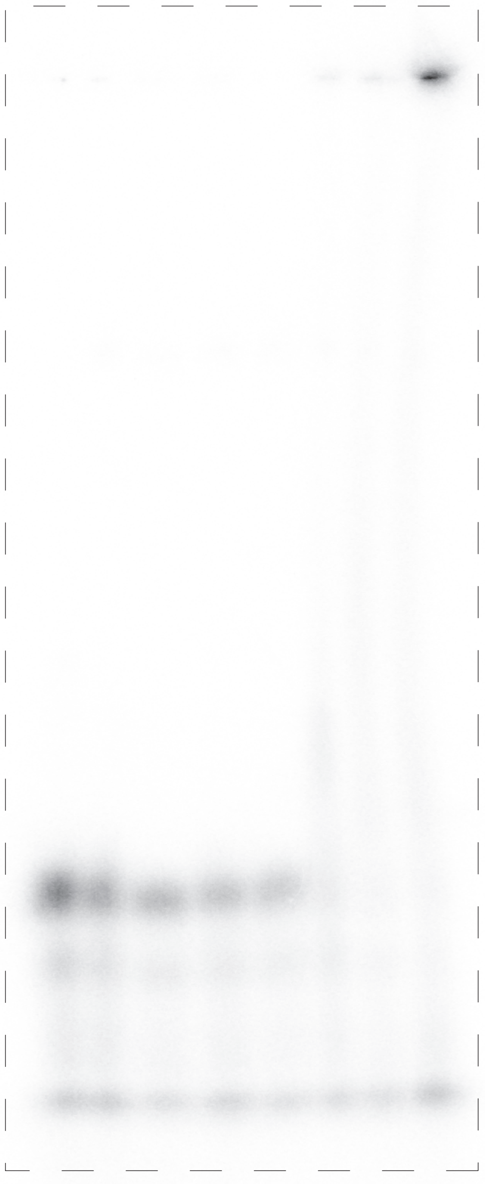

Exteneded data h-1 Raw data

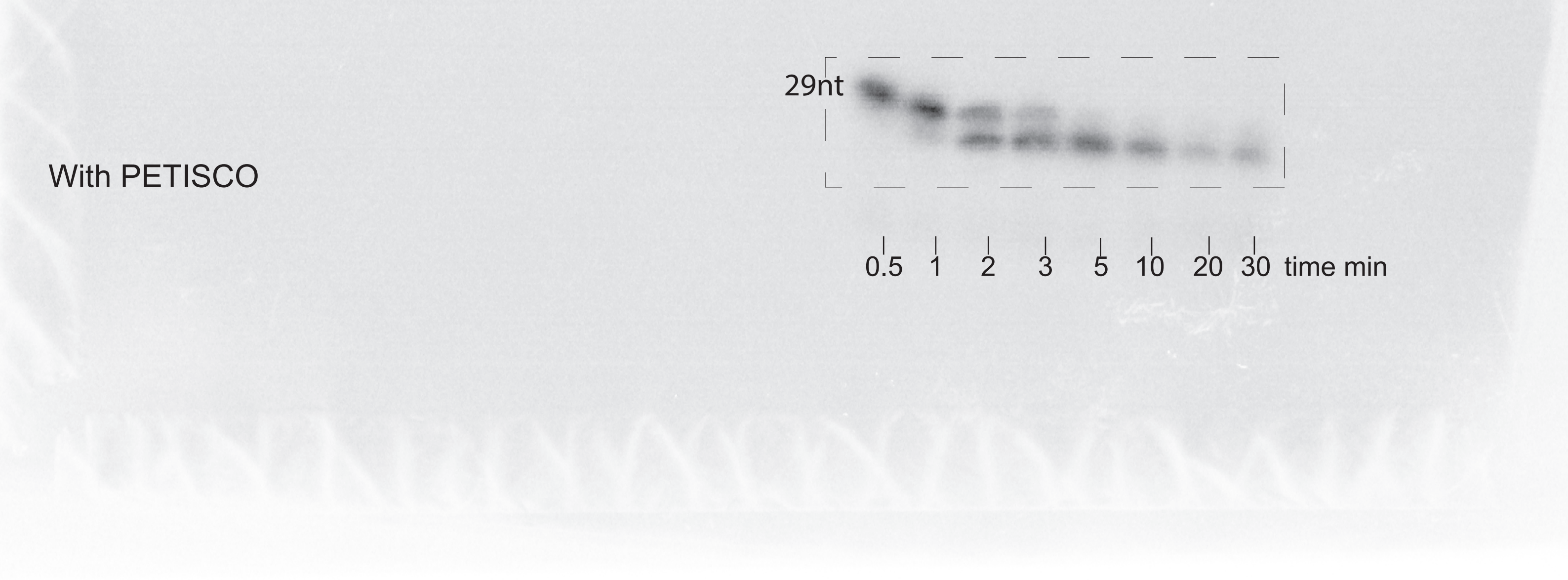

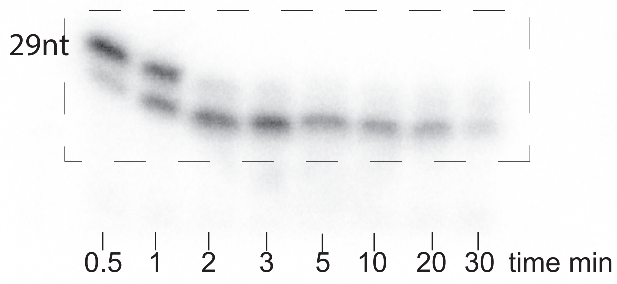

Without PETISCO

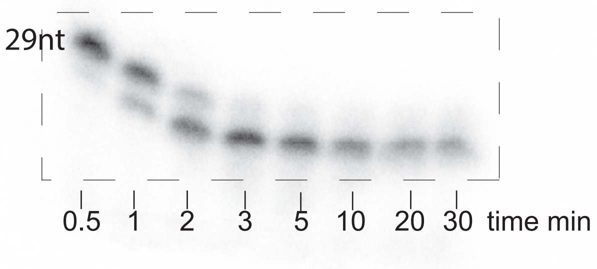

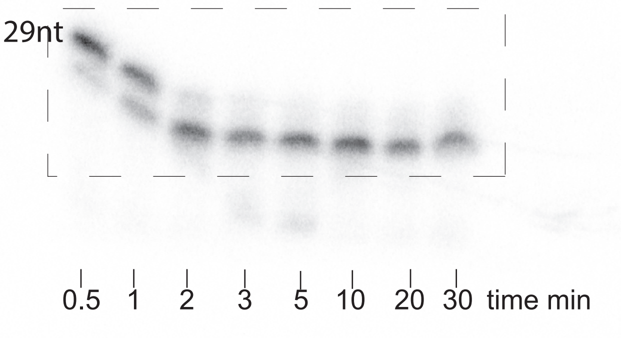

Extended data 7a

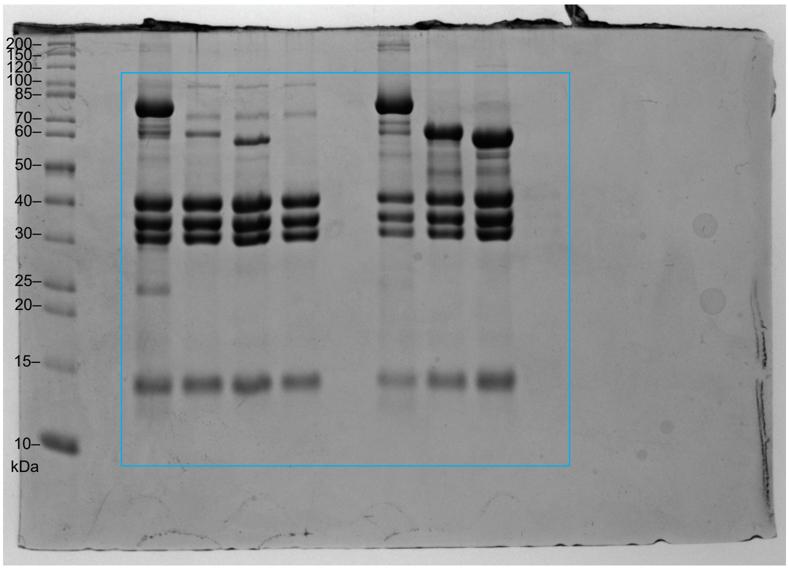

Extended data Figure 7b

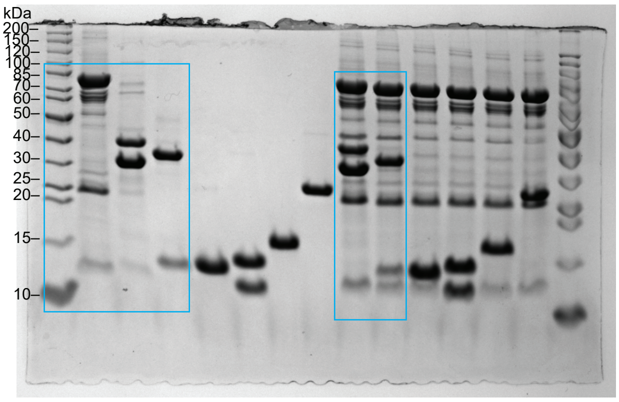

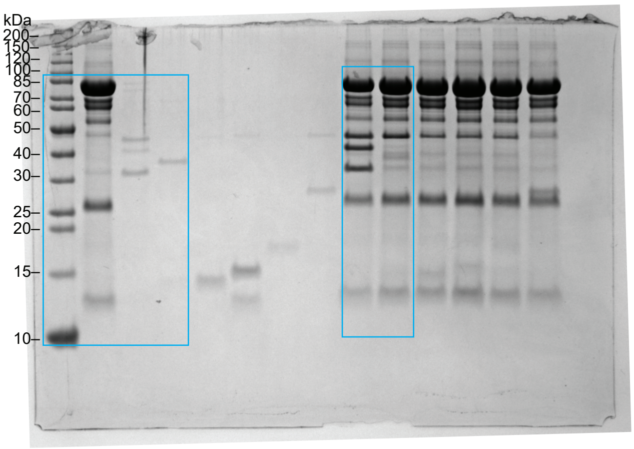

Extended data Figure 7c

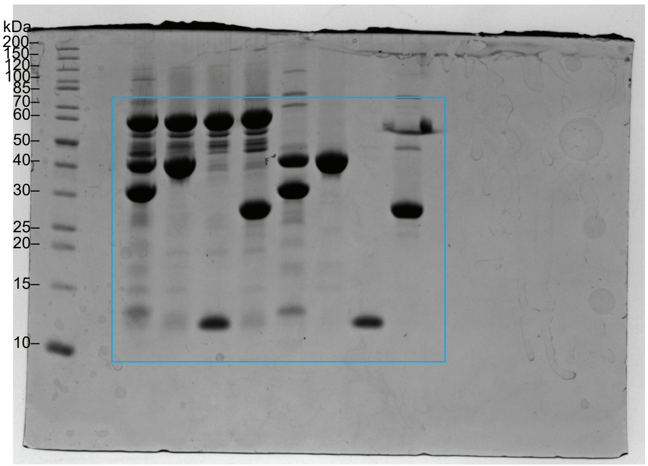

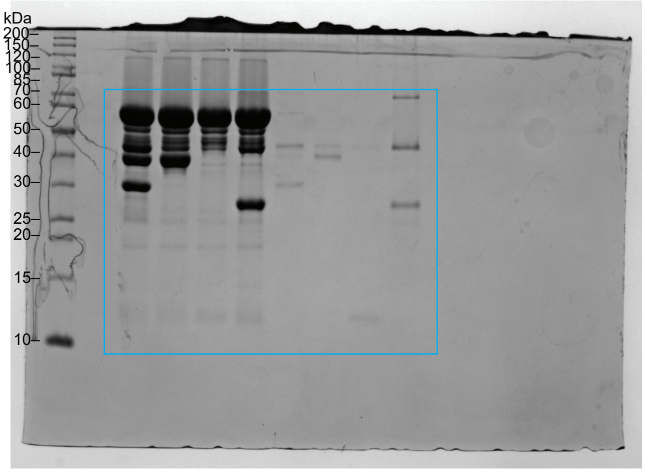

Extended data Figure 7d/e

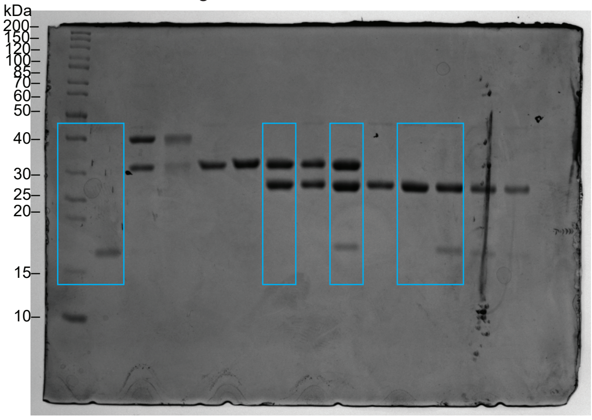

Extended data Figure 9a

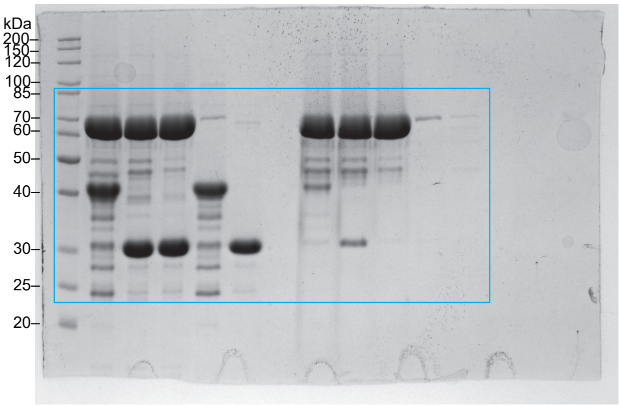

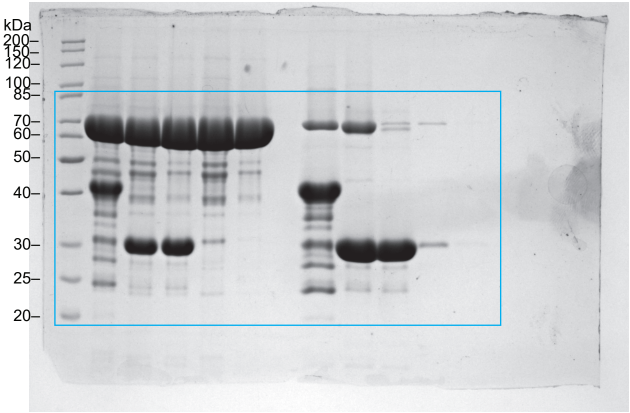

Supplement: Supplementary file 1 — All gels in uncropped format, including annotations and information on controls. [file 41586_2023_6588_MOESM1_ESM.pdf]
