# Supplementary material for: piRNA processing by a trimeric Schlafen-domain nuclease
Source: Nature. 2023 Sep 27;622(7982):402–9. doi: 10.1038/s41586-023-06588-2 (PMC10567574; doi:10.1038/s41586-023-06588-2)
Supplement: Supplementary file 2 — Reporting Summary [file 41586_2023_6588_MOESM2_ESM.pdf]

nature portfolio

Corresponding author(s): Ketting and Falk

Last updated by author(s): 18/08/23

# Reporting Summary

Nature Portfolio wishes to improve the reproducibility of the work that we publish. This form provides structure for consistency and transparency in reporting. For further information on Nature Portfolio policies, see our Editorial Policies and the Editorial Policy Checklist.

## Statistics

For all statistical analyses, confirm that the following items are present in the figure legend, table legend, main text, or Methods section.

| n/a | Confirmed | |
|---|---|---|
| ☐ | ☒ | The exact sample size (*n*) for each experimental group/condition, given as a discrete number and unit of measurement |
| ☐ | ☒ | A statement on whether measurements were taken from distinct samples or whether the same sample was measured repeatedly |
| ☐ | ☒ | The statistical test(s) used AND whether they are one- or two-sided<br>*Only common tests should be described solely by name; describe more complex techniques in the Methods section.* |
| ☒ | ☐ | A description of all covariates tested |
| ☒ | ☐ | A description of any assumptions or corrections, such as tests of normality and adjustment for multiple comparisons |
| ☐ | ☒ | A full description of the statistical parameters including central tendency (e.g. means) or other basic estimates (e.g. regression coefficient) AND variation (e.g. standard deviation) or associated estimates of uncertainty (e.g. confidence intervals) |
| ☐ | ☒ | For null hypothesis testing, the test statistic (e.g. *F*, *t*, *r*) with confidence intervals, effect sizes, degrees of freedom and *P* value noted<br>*Give P values as exact values whenever suitable.* |
| ☒ | ☐ | For Bayesian analysis, information on the choice of priors and Markov chain Monte Carlo settings |
| ☒ | ☐ | For hierarchical and complex designs, identification of the appropriate level for tests and full reporting of outcomes |
| ☒ | ☐ | Estimates of effect sizes (e.g. Cohen's *d*, Pearson's *r*), indicating how they were calculated |

*Our web collection on statistics for biologists contains articles on many of the points above.*

## Software and code

Policy information about availability of computer code

| | |
|---|---|
| Data collection | Unicorn7 software for chromatography<br>MicroCal PEAQ-ITC Control Software v1.41 to acquire ITC data<br>DM6000B microscope: LAS AF 3.1.0 8587<br>SP5 microscope: LAS AF 2.7.3.9723<br>Typhoon FLA 9500 Version V.0 Build 1.0.0.185<br>Image Lab 6.0.1 for Western blots and genotyping gels |
| Data analysis | The following software versions used for analysis:<br>All computational tools that were used are public and details on their use are provided in the Methods section. No custom algorithms or software were developed.<br>FastQC v.0.11.9<br>MultiQC v.1.9<br>Cutadapt v.4.0<br>Bowtie v.1.3.1<br>Samtools v.1.10<br>GNU Awk v.5.1.0<br>Subread v.1.6.2<br>Bedtools v.2.27.1<br>kentUtils v.385<br>IGV v.2.15.4<br>R v.4.1.0 and v5 |

Phenix v.1.20.1-4487
ChimeraX v.1.5
autoPROC v.1.1.7
XDS VERSION Feb 5, 2021
AIMLESS v. 0.7.7
COOT v0.9.8.6
MaxQuant suite 1.6.5.0
Adobe Illustrator 2023
ImageJ 64 V5
Datagraph5

For manuscripts utilizing custom algorithms or software that are central to the research but not yet described in published literature, software must be made available to editors and reviewers. We strongly encourage code deposition in a community repository (e.g. GitHub). See the Nature Portfolio guidelines for submitting code & software for further information.

## Data

Policy information about availability of data

All manuscripts must include a data availability statement. This statement should provide the following information, where applicable:

- Accession codes, unique identifiers, or web links for publicly available datasets
- A description of any restrictions on data availability
- For clinical datasets or third party data, please ensure that the statement adheres to our policy

Sequencing data is available at NCBI's Sequence Read Archive under accession number PRJNA925182 (https://dataview.ncbi.nlm.nih.gov/object/PRJNA925182?reviewer=951lavsn8a0umpj5j17m8bk738).
The mass spectrometry proteomics data have been deposited to the ProteomeXchange Consortium via the PRIDE72 partner repository with the dataset identifier PXD039502. Username: reviewer_pxd039502@ebi.ac.uk
Password: T2C6anBX
Coordinates and structure factors of the TOFU-6eTUDOR TOFU-1pep complex structure have been deposited in the Protein Data Bank with accession codes PDB ID 8BY5.
Wormbase WS289 was used in this study.
Uniprot was regularly used. Because of constant updates specific versions cannot be given. Always the most recent version was used.
Alphafold database (https://alphafold.ebi.ac.uk/) was used in this work.

## Human research participants

Policy information about studies involving human research participants and Sex and Gender in Research.

| | |
|---|---|
| Reporting on sex and gender | NA |
| Population characteristics | NA |
| Recruitment | NA |
| Ethics oversight | NA |

Note that full information on the approval of the study protocol must also be provided in the manuscript.

# Field-specific reporting

Please select the one below that is the best fit for your research. If you are not sure, read the appropriate sections before making your selection.

☒ Life sciences ☐ Behavioural & social sciences ☐ Ecological, evolutionary & environmental sciences

For a reference copy of the document with all sections, see nature.com/documents/nr-reporting-summary-flat.pdf

# Life sciences study design

All studies must disclose on these points even when the disclosure is negative.

| | |
|---|---|
| Sample size | Sample sizes for the worm experiments were not based on statistical methods but on previously published similar experiments yielding consistent and reproducible results. For small RNA sequencing 3 biological replicates yield very robust results.<br>Sample sizes for mass spectrometry experiments were established as quadruplicates (LFQ), as is well-accepted in the proteomics field. The four samples were biological replicates. For immunoprecipitation and Western Blot experiments, animals were prepared from bleaching 1-2 high density plates of gravid adults per sample, L4 samples, young adult samples, and gravid adult samples were prepared from a pool of synchronized animals (200 µl per sample). All blots and IPs were done in two independent biological experiments. |
| Data exclusions | No data was excluded |

| | |
|---|---|
| Replication | All gels and blots were done in duplicate.<br>All protein analyses were done in duplicate.<br>Sequencing data were obtained from one experiment based on biological triplicates. In our experience this yields very robust results. Many papers build on duplicates only. The tofu-2(e216a) mutant was analyzed twice in this manner.<br>Mass spectrometry is based on one experiment that uses biological quadruplicates. This is widely accepted practice in the field of quantitative proteomics.<br>Imaging results were based on observations on different individuals animals. At least ten animals were imaged and a representative image was used in the manuscript. Images of cells were obtained with two different transfection experiments. Representative images of cells are shown in the manuscript. Seven cells were imaged in both experiments.<br>All PUCH cleavage reactions were done in duplicate. The ligation experiment was done in triplicate. Cleavage of the 10 nucleotide substrate and the gel-shift with the 5'P end were the only experiments performed once.<br>In all cases replication yielded equivalent results. |
| Randomization | Randomization is not relevant to this study, as distinct genotypes and protein preparations had to be generated before each experiment. |
| Blinding | Blinding was used in the initial analysis of the sRNA data. The bio-informatician received only strain names without genotype information. After initial analysis, results were interpreted in light of the genotypes, so at this stage no blinding was applied. This is also not possible if questions are derived from the initial results of analysis.<br>Blinding was applied in the in vitro PUCH activity assays from lysates of transfected BmN4 cells. Lysate sources were unknown to the N. Podvalnaya when running the cleavage assays.<br>In all other experiments blinding was not used in experiments for the following reasons:<br>The primary results of our work were rather objective, making blinding not needed.<br>The explorative nature of the study makes blinding impossible and rather unlikely to affect the results.<br>Genotypes of animals needed to be established before analysis. |

# Reporting for specific materials, systems and methods

We require information from authors about some types of materials, experimental systems and methods used in many studies. Here, indicate whether each material, system or method listed is relevant to your study. If you are not sure if a list item applies to your research, read the appropriate section before selecting a response.

## Materials & experimental systems

| n/a | Involved in the study | |
|---|---|---|
| ☐ | ☒ | Antibodies |
| ☐ | ☒ | Eukaryotic cell lines |
| ☒ | ☐ | Palaeontology and archaeology |
| ☐ | ☒ | Animals and other organisms |
| ☒ | ☐ | Clinical data |
| ☒ | ☐ | Dual use research of concern |

## Methods

| n/a | Involved in the study | |
|---|---|---|
| ☒ | ☐ | ChIP-seq |
| ☒ | ☐ | Flow cytometry |
| ☒ | ☐ | MRI-based neuroimaging |

## Antibodies

| | |
|---|---|
| Antibodies used | Monoclonal anti-HA (clone 12CA5) mouse antibody, in-house production; 1:1,000<br>anti-Histone H3, Art. No. H0164, Sigma-Aldrich; 1:1,000<br>anti-mouse IgG, HRP-linked antibody, Art. No. 7076, Cell Signaling Technology; 1:10,000<br>anti-rabbit IgG, HRP-linked antibody, Art. No. 7074, Cell Signaling Technology; 1:10,000<br>Monoclonal anti-MYC (clone 9B11) mouse antibody, #2276S, Cell Signaling; 1:1,000<br>Anti-Actin polyclonal antibody raised in Rabbit (Sigma, #A5060); 1:1,000<br>IRDye® 800CW Goat anti-Mouse IgG Secondary Antibody (LI-COR, #926-32210); 1:10,000<br>IRDye® 680LT Donkey anti-Rabbit IgG (LI-COR, #926-68023); 1:10,000<br>Monoclonal ANTI-FLAG® (clone M2), Art. No. F3165, Sigma-Aldrich; 1:1,000<br>Monoclonal anti-HA (clone 12CA5) mouse antibody, in-house production; 1:1,000<br>Monoclonal Anti-GFP Antibody (clone B-2), Santa Cruz, Cat. #sc-9996, Lot.#K1115; 1:1,000 |
| Validation | BmN4 cell work:<br>Absence of signal in absence of expression of tagged protein was taken as verification of specificity in all cases.<br>Anti-Actin (Sigma, #A5060). Quality control by Sigma-Aldrich: working dilutions for western blot of at least 1:250 were determined using rat brain or chicken muscle extracts<br><br>IRDye® 800CW Goat anti-Mouse IgG Secondary Antibody (LI-COR, #926-32210): Isolation of specific antibodies was accomplished by affinity chromatography using pooled mouse IgG covalently linked to agarose. Based on ELISA and flow cytometry, this antibody reacts with the heavy and light chains of mouse IgG1, IgG2a, IgG2b, and IgG3, and with the light chains of mouse IgM and IgA. This antibody was tested by dot blot and and/or solid-phase adsorbed for minimal cross-reactivity with human, rabbit, goat, rat, and horse serum proteins, but may cross-react with immunoglobulins from other species. The conjugate has been specifically tested and qualified for Western blot applications. |

IRDye® 680LT Donkey anti-Rabbit IgG (LI-COR, #926-68023). The antibody was isolated from antisera by immunoaffinity chromatography using antigens coupled to agarose beads. Based on immune electrophoresis, this antibody reacts with the heavy chains of rabbit IgG, and with the light chains common to most rabbit immunoglobulins. No reactivity was detected against non-immunoglobulin serum proteins. This antibody was tested by ELISA and/or solid-phase adsorbed to ensure minimal cross-reactivity with bovine, chicken, goat, guinea pig, Syrian hamster, horse, human, mouse, rat, and sheep serum proteins, but may cross-react with immunoglobulins from other species. The conjugate has been specifically tested and qualified for Western blot applications.

Worm work:
Specificity tested by absence of signal in lysates from untagged strains.
Monoclonal anti-HA (clone 12CA5) antibody: Soluble lysate from exponentially-growing yeast cultures expressing HA-tagged proteins were separated on 4-15% gradient gel (BioRad). Proteins were blotted an a nitrocellulose membrane using semi-dry transfer (10 min High MW program on TransBlot Turbo blotter) and the membrane was blocked 1h in 5% skim milk/PBS/0.1% Tween-20. Primary antibodies: mouse anti-HA (in-house or Covance, 1:1000 o/N in blocking solution) at 4°C Secondary antibody: goat anti-mouse HRP-coupled (BIORAD 170-5047) 1:3000 1h at RT in blocking solution. The western blot was developed using ECL substrate Dura (Thermo, #34076). Exposure times as indicated in the figure. The experiment was performed by Katharina Bender from Brian Luke's group, IMB, Mainz, Germany.
anti-Histone H3 antibody: By immunoblotting, a working antibody dilution of 1:5,000-1:10,000 is recommended using a whole cell extract of the A431 human epidermoid carcinoma cell line, and a whole cell extract of the mouse fibroblast NIH3T3 cell line. By immunoblotting, a working antibody dilution of 1:2,500-1:5,000 is recommended using a whole cell extract of the rat pheochromocytoma PC12 cell line.
anti-mouse IgG, HRP-linked antibody: Application Key: WB-Western Blot IP-Immunoprecipitation IHC-Immunohistochemistry ChIP-Chromatin Immunoprecipitation IF-Immunofluorescence F-Flow Cytometry E-P-ELISA-Peptide. Species Cross-Reactivity Key: H-Human M-Mouse R-Rat Hm-Hamster Mk-Monkey Vir-Virus Mi-Mink C-Chicken Dm-D. melanogaster X-Xenopus Z-Zebrafish B-Bovine Dg-Dog Pg-Pig Sc-S. cerevisiae Ce-C. elegans Hr-Horse All-All Species Expected anti-rabbit IgG, HRP-linked antibody: Application Key: WB-Western Blot IP-Immunoprecipitation IHC-Immunohistochemistry ChIP-Chromatin Immunoprecipitation IF-Immunofluorescence F-Flow Cytometry E-P-ELISA-Peptide. Species Cross-Reactivity Key: H-Human M-Mouse R-Rat Hm-Hamster Mk-Monkey Vir-Virus Mi-Mink C-Chicken Dm-D. melanogaster X-Xenopus Z-Zebrafish B-Bovine Dg-Dog Pg-Pig Sc-S. cerevisiae Ce-C. elegans Hr-Horse All-All Species Expected
Monoclonal anti-MYC: no signal in non-tagged C. elegans strains; signal at expected MW in tagged strains only.

## Eukaryotic cell lines

Policy information about cell lines and Sex and Gender in Research

| | |
|---|---|
| Cell line source(s) | BmN4 cells used in this study were provided to us by Dr. Ramesh Pillai, University of Geneva, in 2015. BmN4 cells originate from Kyushu University, Dr. Kusakabe. Also see: https://www.cellosaurus.org/CVCL_Z634 |
| Authentication | BmN4 cells have not been authenticated. |
| Mycoplasma contamination | Not tested. |
| Commonly misidentified lines (See ICLAC register) | No commonly misidentified cell lines were used. |

## Animals and other research organisms

Policy information about studies involving animals; ARRIVE guidelines recommended for reporting animal research, and Sex and Gender in Research

Laboratory animals

In all experiments young adult animals were used.
Caenorhabditis elegans strains were all based on the wild-type isolated Bristol N2, which was used as control in many experiments.
Strains developed and/or used in this study:
RFK1269 tofu-2(xf245[tofu-2::HA]), V.
RFK1273 tofu-2(xf246[E216A]::HA]), V.
RFK1059 tofu-2(xf231[E216A]]) V.
RFK1242 pid-1(xf35) II; tofu-2(xf231) V.
RFK1095 mjSi22 [Pmex-5::mCherry::his-58::21UR-1_as::tbb-2(3'UTR)] I, tofu-2(xf231) V.
RFK1246 mjSi22 [mex-5p::mCherry::his-58 + 21UR-1_as + tbb-2(3'UTR)] I; mut-7(xf125) III.
RFK851 mjSi22 [Pmex-5::mCherry::his-58::21UR-1_as::tbb-2(3'UTR)] I prg-1(n4357) I.
RFK204 mjSi22 [Pmex-5::mCherry::his-58::21UR-1_as::tbb-2(3'UTR)] I.
RFK1481 slfl-3(xf248) I.
RFK1580 tofu-1(xf337[L88R;L92R]) V.
RFK1506 tofu-6(xf312[V266E]::3xMYC) I.
RFK1605 tofu-1(xf337) V; tofu-6(xf312) I.
RFK1057 tofu-6(xf229[tofu-6::3xMYC]) II.
RFK1639 slfl-4(xf351) IV.
RFK1640 slfl-3(xf248) I, slfl-4(xf351) IV.
RFK1689 slfl-3(xf356) I; slfl-4(xf351) IV.
RFK1692 tofu-1(xf358[tofu-1::3MYC]), V.
RFK1693 tofu-1(xf363[3MYC::tofu-1[L88R&L92R]]) V.

Wild animals

No wild animals were used in this study

| | |
|---|---|
| Reporting on sex | All studies used hermaphrodites. Males occur spontaneously in cultures, but only at a low frequency, and for these studies can be neglected. |
| Field-collected samples | No filed-collected samples were used in this study |
| Ethics oversight | This study did nor require ethical approval |

Note that full information on the approval of the study protocol must also be provided in the manuscript.
